# Sex disparities in physical activity domains and hypertension prevalence

**DOI:** 10.1186/s40885-023-00260-7

**Published:** 2024-01-02

**Authors:** Min Jeong Cho, Yong Joon Jung, Ho Jeong Min, Hyun Jeong Kim, Setor K. Kunutsor, Sae Young Jae

**Affiliations:** 1 Department of Sport Science, University of Seoul, Seoul, Republic of Korea; 2 Diabetes Research Centre, Leicester General Hospital, University of Leicester, Leicester, UK; 3 Graduate School of Urban Public Health, University of Seoul, Seoul, Republic of Korea

**Keywords:** Hypertension, Leisure time physical activity, Occupational physical activity

## Abstract

**Background:**

This study aimed to examine the associations of leisure time physical activity (LTPA) and occupational physical activity (OPA) with the prevalence of hypertension, while exploring the sex disparities in these associations.

**Methods:**

A cross-sectional study was conducted using data from the Korea National Health and Nutrition Examination Survey between 2014 and 2019 (n = 26,534). Hypertension was defined as the use of antihypertensive drugs or systolic and diastolic blood pressure ≥ 140/90 mm Hg. Self-reported physical activity (PA), assessed by the global PA questionnaire, was categorized into three domains: total PA, LTPA and OPA. Each PA domain was classified based on METs-min/wk and intensity.

**Results:**

In a multivariable adjusted model, the odds ratio (OR) with 95% confidence intervals (CIs) for the prevalence of hypertension in the active versus inactive group, based on METs, was 0.92 (95% CI 0.85–0.99) for total PA, 0.90 (95% CI 0.83–0.98) for LTPA and 1.21 (95% CI 1.05–1.38) for OPA. Compared to the inactive group, moderate to vigorous intensity was associated with a lower odds of hypertension for total PA and LTPA (total PA: OR 0.95, 95% CI 0.89-1.00 and LTPA: OR 0.92, 95% CI 0.86–0.98), but a higher odd for OPA (OR 1.17, 95% CI 1.05–1.30). Subgroup analyses showed significant evidence of effect modification by sex on the associations of total PA and LTPA (METs and intensity) with hypertension prevalence (*p*-values for interaction < 0.01); the associations were generally stronger for women. OPA was associated with a higher prevalence of hypertension in women, but not in men (*p*-value for interaction > 0.05).

**Conclusions:**

Higher levels of total PA and LTPA were associated with lower prevalence of hypertension in both men and women, with slightly stronger associations for women. However, higher OPA was associated with a higher prevalence of hypertension in women. These findings support the PA health paradox hypothesis and highlight the sex disparities in the association between OPA and hypertension prevalence.

## Background

Hypertension, a global public health concern associated with cardiovascular disease (CVD) and premature death [1, 2], is influenced by multiple risk factors including age, unhealthy lifestyle, environmental factors, and psychological stress [2]. Physical activity (PA), regarded as a vital component of a healthy lifestyle, is widely acknowledged for its role in the prevention and management of CVDs. However, recent studies suggest that the relationship between PA and cardiovascular outcomes differs based on the specific domain of PA [3].

Leisure time physical activity (LTPA) is associated with a lower risk of cardiovascular mortality [4], while occupational physical activity (OPA) has shown associations with adverse cardiovascular outcomes [5–7], leading to what is known as the PA health paradox [3]. However, the contradictory findings concerning different PA domains are not consistently observed [8, 9]. Consequently, the contrasting health benefits of LTPA and OPA have sparked a scientific debate. Evaluating this phenomenon may offer insights into the variations observed in the effects of LTPA and OPA.

It has been reported that the association between OPA and cardiovascular risk may vary by sex [10, 11]. However, the impact of different PA domains on hypertension risk sensitivity remains unclear, despite the well-established benefits of LTPA on cardiovascular health. While some studies have indicated a dose-response association between LTPA and a lower risk of hypertension [12, 13], research on OPA and hypertension has yielded mixed results. Certain studies have shown that higher OPA is associated with an increased risk of hypertension [14, 15], but these findings are not consistent across all studies [12, 16]. Alternatively, some studies have reported an inverse association between OPA and hypertension [17–19], while others have observed a U-shaped relationship [20]. As a result, the association between OPA and hypertension remains conflicting, necessitating further studies to evaluate the relationship between OPA, including intensity and volume variables, and the risk of hypertension.

Furthermore, the impact of sex differences on the sensitivity of OPA to cardiovascular outcomes is a subject of ongoing debate [10, 11, 21] and the results in this regard are inconclusive. The existence of sex differences in the association between OPA and hypertension remains uncertain. Therefore, it would be interesting to explore whether the association between OPA and hypertension varies by sex.

This study aimed to investigate the association between PA domains and the prevalence of hypertension in Korean adults. Furthermore, we explored potential sex differences in the association between OPA and hypertension. We hypothesized that higher LTPA would be associated with a lower prevalence of hypertension, while higher OPA would be associated with a higher prevalence of hypertension.

## Methods

### Study participants

**Fig. 1:**
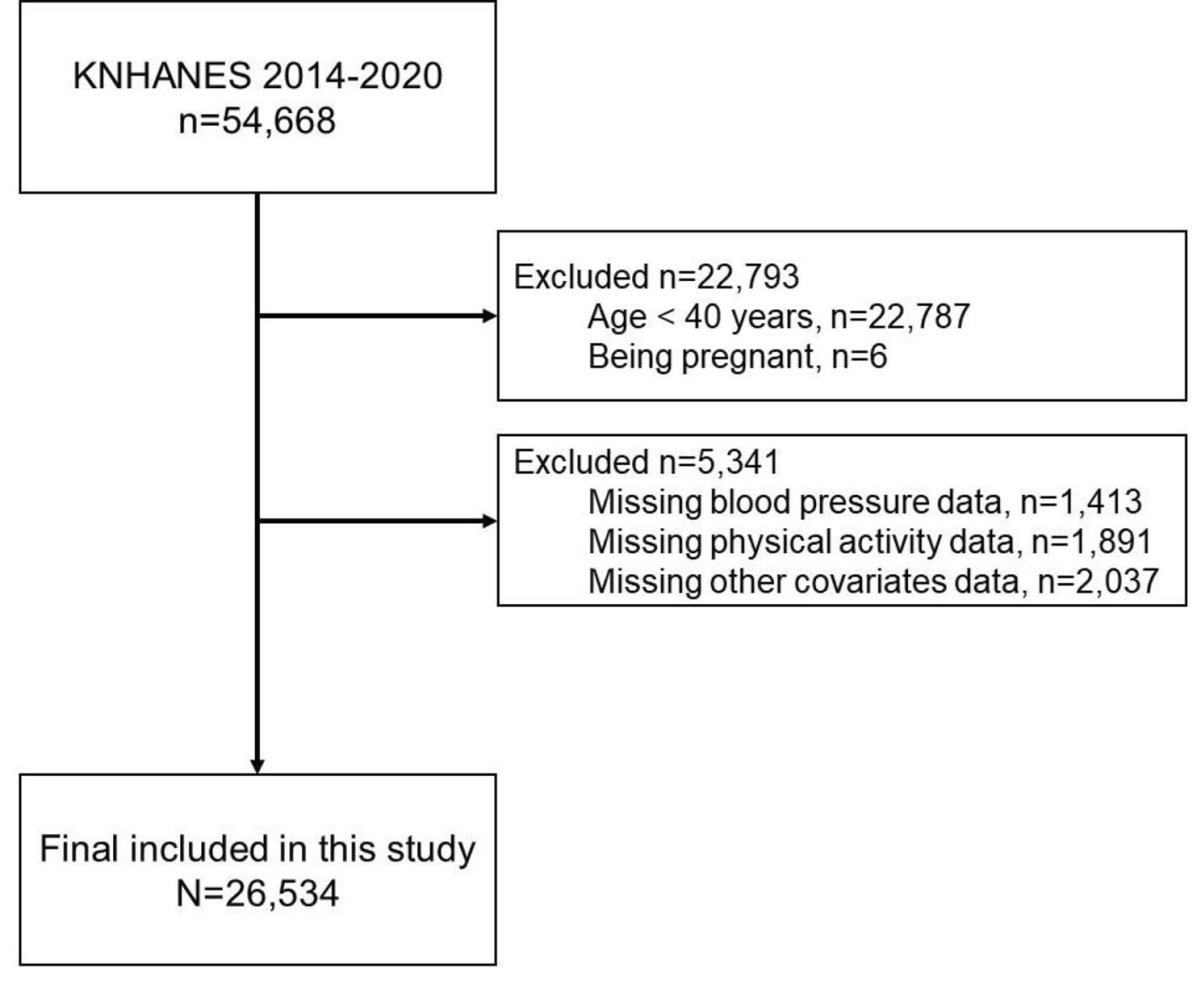
Flow chart of study participants

This cross-sectional study utilized data collected from the Korean National Health and Nutrition Survey (KNHANES) spanning the years 2014 to 2020. The KNHANES, conducted by the Korea Disease Control and Prevention Agency of the Ministry of Health and Welfare, involved health, nutrition, and screening surveys administered to representative samples of residents in Korea annually. Data were obtained through health questionnaires, interviews, physical measurements, and clinical tests. Out of the initial 54,668 individuals invited to participate, we excluded 22,787 participants who were less than 40 years of age and pregnant (n = 6). In addition, we excluded 5,341 participants who did not have data on blood pressure, PA, and other covariates factors. Ultimately, a total of 26,534 participants with complete data on blood pressure, PA, and covariates factors were included in the analysis. The flowchart of the analytic sample is described in Fig. 1. Ethical approval for this study was obtained from the Institutional Review Board of the University of Seoul (IRB File No. 2021-10-003).

### Outcome variables

The prevalence of hypertension was defined as the number of participants having systolic blood pressure (SBP) ≥ 140 mmHg or diastolic blood pressure (DBP) ≥ 90 mmHg or currently taking antihypertensive medication during the survey period.

### Exposure variables

#### Physical activity

The global PA questionnaire (GPAQ) developed by the WHO, was used to assess PA in the KNHANES. The GPAQ categorized PA into three domains: LTPA, transport PA (TPA), and OPA. LTPA and OPA consisted of 6 questions each, while TPA included 3 questions. Participants were queried about the types of activities performed, the number of days per week engaged in activity, and the duration in hours and minutes of daily activity. Vigorous exercise was defined as high-intensity sport, athletics, and recreational activities that substantially elevated heart rate and caused breathlessness for at least 10 min (e.g., running, jumping rope, mountain climbing, basketball, swimming, badminton singles, etc.). Moderate exercise was defined as moderate-intensity sports or activities that led to a slightly increased heartbeat and mild shortness of breath for at least 10 min^,^ (e.g., brisk walking, jogging, weight training, golf, dance sports, pilates, etc.). PA was categorized into three domains: total PA, LTPA and OPA. Each PA domain was classified into 3 or 2 groups according to the total volume of METs and the intensity of PA, respectively. Each PA domain was classified according to METs as inactive (0 METs-min/wk), insufficiently active (1-499 METs-min/wk), and active (≥ 500 METs-min/wk). Intensity was categorized as either inactive or moderate to vigorous intensity.

#### Covariates

The level of education, a component of socioeconomic status, was categorized into four groups: elementary school graduates, middle school graduates, high school graduates, and college graduates or higher. Hypertension was defined as having a systolic blood pressure of ≥ 140mmHg, a diastolic blood pressure of ≥ 90mmHg, or the use of antihypertensive medications. Diabetes was defined as a fasting glucose level of ≥ 126 mg/dL, the use of oral hypoglycemic agents or insulin, or a diagnosis by a physician. Obesity was defined as a body mass index (BMI) of ≥ 25 kg/m^2^, calculated as body weight in kilograms divided by the square of height in meters.

Household income was divided into quartiles based on the monthly average household equalization income, categorized as lower, lower-middle, upper-middle, and upper classes. Alcohol consumption was classified into drinking and non-drinking groups. Individuals who consumed alcohol more than once a month in the past year were included in the drinking group, while those who consumed alcohol less than one time or abstained completely were classified as the non-drinking group. Smoking status was categorized into smoking and non-smoking groups. The smoking group included individuals who had smoked more than five packs of cigarettes throughout their lifetime and were currently smoking, while the non-smoking group consisted of individuals who had smoked less than five packs throughout their lifetime or had never smoked.

### Statistical analysis

To compare the characteristics of participants with and without hypertension, we used the complex sample chi-square test for categorical variables and the complex sample t-test for continuous variables. We calculated odds ratios (OR) and 95% confidence intervals (CI) from logistic regression analyses with adjustment for confounding factors (age, sex, obesity, smoking, alcohol consumption, total cholesterol, glucose, and education, and LTPA when exposed OPA or OPA when exposed LTPA) to determine the associations of PA domains (METs and intensity) with the prevalence of hypertension. We used interaction tests to assess for statistical evidence of effect modification by sex. We used receiver operating characteristic (ROC) to determine the best cutoff point of total physical activity levels which could be used to predict prevalent hypertension, with the predictive accuracy expressed as area under curve (AUC). All analyses were conducted using SPSS (IBM Corp., SPSS Version 26.0, Armonk, NY, USA), and alpha was set at p < 0.05.

## Results

Table 1 provides an overview of the characteristics of the study participants with and without hypertension. The prevalence of hypertension was 44.7% among men and 38.3% among women. Participants with hypertension were generally older, more likely to have diabetes and obesity, and had a higher prevalence of smoking. They also exhibited significantly lower alcohol consumption and lower socioeconomic status compared to participants without hypertension.

**Table 1:** Characteristics of the participants overall and by hypertension (HTN) status (n = 26,534)

Characteristics	Categories	Overall n (%)	Non-HTN n (%)	HTN n (%)	χ^2^ (*p*)
Sex	Men	11,501 (43.3)	6357 (55.3)	5144 (44.7)	112.733 (< 0.001)
	Women	15,033 (56.7)	9282 (61.7)	5751 (38.3)	
Age	40–49	6727 (25.4)	5474 (81.4)	1253 (18.6)	3382.61 (< 0.001)
	50–59	7213 (27.2)	4819 (66.8)	2394 (33.2)	
	60–69	6691 (25.2)	3380 (50.5)	3311 (49.5)	
	≥ 70	5903 (22.2)	1966 (33.3)	3937 (66.7)	
Household income	1st quartile (lowest)	5864 (22.1)	2452 (41.8)	3412 (58.2)	1161.16 (< 0.001)
	2nd quartile	6627 (25.0)	3738 (56.4)	2889 (43.6)	
	3rd quartile	6737 (25.4)	4377 (65.0)	2360 (35.0)	
	4th quartile (highest)	7306 (27.5)	5072 (69.4)	2234 (30.6)	
Education	Elementary school	7185 (27.1)	2861 (39.8)	4324 (60.2)	1866.61 (< 0.001)
	Middle school	3584 (13.5)	1925 (53.7)	1659 (46.3)	
	High school	8302 (31.3)	5404 (65.1)	2898 (34.9)	
	≥College	7463 (28.1)	5449 (73.0)	2014 (27.0)	
Diabetes mellitus	No	22,140 (83.4)	14,029 (63.4)	8111 (36.6)	1081.96 (< 0.001)
	Yes	4394 (16.6)	1610 (36.6)	2784 (63.4)	
Obesity	No	20,614 (77.7)	12,925 (62.7)	7689 (37.3)	539.93 (< 0.001)
	Yes	5920 (22.3)	2714 (45.8)	3206 (54.2)	
Smoking	No	15,861 (59.8)	9623 (60.7)	6238 (39.3)	48.84 (< 0.001)
	Yes	10,673 (40.2)	6016 (56.4)	4657 (43.6)	
Alcohol consumption	No	3717 (14.0)	1786 (48.0)	1931 (52.0)	211.82 (< 0.001)
	Yes	22,817 (86.0)	13,853 (60.7)	8964 (39.3)	
Total physical activity	Inactive	9277 (35.0)	5052 (54.5)	4225 (45.5)	208.49 (< 0.001)
	Insufficiently active	11,028 (41.6)	6471 (58.7)	4557 (41.3)	
	Active	6229 (23.3)	4116 (66.1)	2113 (33.9)	
Leisure time physical activity	Inactive	19,795 (74.6)	11,106 (56.1)	8689 (43.9)	258.91 (< 0.001)
	Insufficiently active	2620 (9.9)	1754 (66.9)	866 (33.1)	
	Active	4119 (15.5)	2779 (67.5)	1340 (32.5)	
Occupational physical activity	Inactive	24,743 (93.3)	14,526 (58.7)	10,217 (41.3)	8.23 (0.016)
	Insufficiently active	629 (2.4)	388 (61.7)	241 (38.3)	
	Active	1162 (4.4)	725 (62.4)	437 (37.6)	

**Fig. 2:**
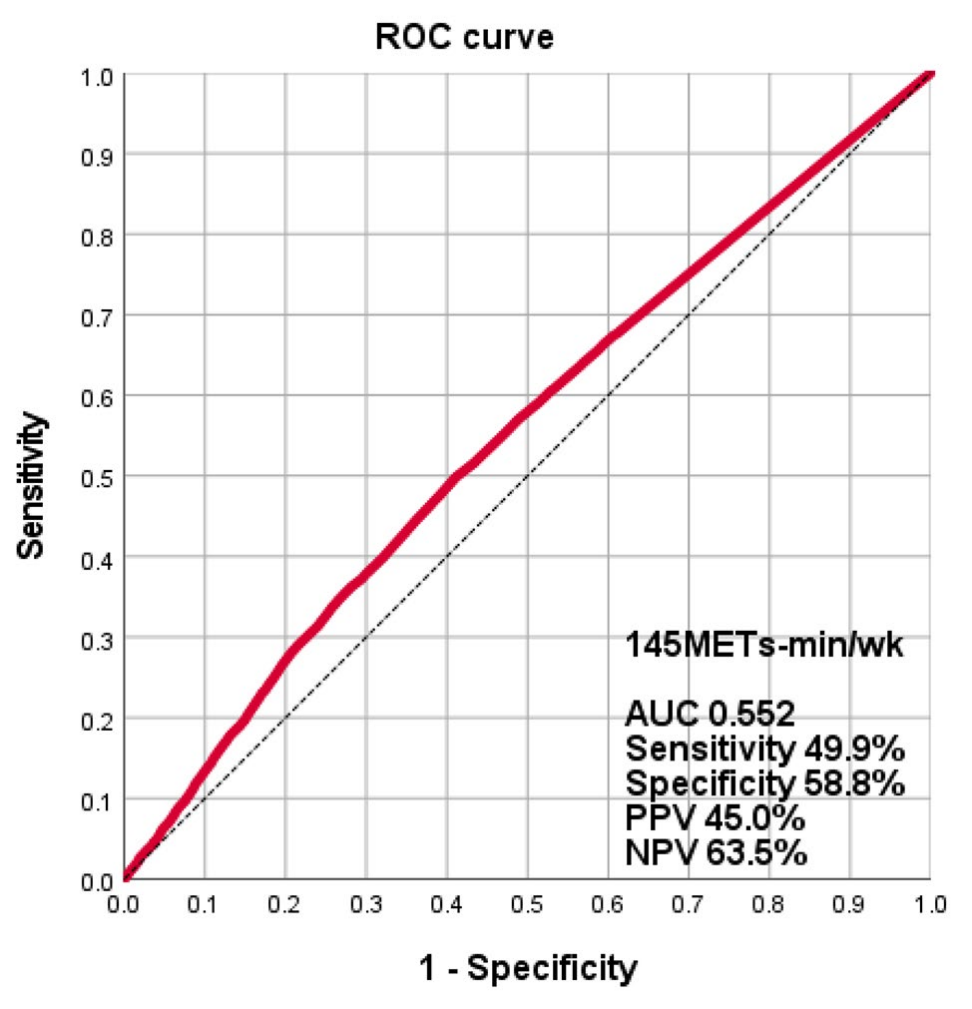
Receiver operating characteristic (ROC) curve for predicting hypertension with total physical activity level. AUC, area under curve; NPV, negative predictive value; and PPV, positive predictive value

When considering total PA, participants classified as active had a significantly lower prevalence of hypertension compared to those classified as inactive (33.9% vs. 45.5%, p < 0.01). Furthermore, active LTPA was associated with a significantly lower prevalence of hypertension compared to inactive LTPA (32.5% vs. 43.9%, p < 0.01), while active OPA demonstrated a lower prevalence of hypertension compared to inactive OPA (37.6% vs. 41.3%, p = 0.016). The ROC curve showed that a total physical activity threshold of 145 METs-min/wk had 50% sensitivity and 59% specificity as a predictive marker for prevalent hypertension. The AUC was 0.5520 (Fig. 2).

In the multivariable analysis adjusting for age, sex, obesity, smoking, alcohol consumption, total cholesterol, glucose, education, and either LTPA (with OPA as the exposure) or OPA (with LTPA as the exposure), the OR and 95% CIs for the prevalence of hypertension among the active versus inactive participants were as follows: Total PA - OR 0.92 (0.85–0.99), LTPA - OR 0.90 (0.83–0.98) and OPA - OR 1.21 (95% CI 1.05–1.38). Furthermore, in terms of intensity, moderate to vigorous intensity of total PA and LTPA was associated with a lower prevalence of hypertension (total PA: OR 0.95, 95% CI 0.89-1.00 and LTPA: OR 0.92, 95% CI 0.86–0.98), whereas moderate to vigorous intensity of OPA was associated with a higher prevalence of hypertension (OR 1.17, 95% CI 1.05–1.30) when compared with the inactive group (Table 2).

**Table 2:** Associations of total PA, LTPA and OPA with the prevalence of hypertension in all participants

Variables	n	Prevalence n (%)	Unadjusted OR (95% CI)	Model 1 OR (95% CI)	Model 2 OR (95% CI)	Model 3 OR (95% CI)
**by METs**						
**Total PA**						
Inactive (0 METs-min/wk)	9277	4225 (45.5)	1 (reference)	1 (reference)	1 (reference)	
Insufficiently active (1-499 METs-min/wk)	11,028	4557 (41.3)	0.84 (0.80–0.89)	0.91 (0.86–0.97)	0.96 (0.90–1.02)	
Active (≥ 500 METs-min/wk)	6229	2113 (33.9)	0.61 (0.57–0.66)	0.81 (0.76–0.87)	0.92 (0.85–0.99)	
**LTPA**						
Inactive (0 METs-min/wk)	19,795	8689 (43.9)	1 (reference)	1 (reference)	1 (reference)	1 (reference)
Insufficiently active (1-499 METs-min/wk)	2620	866 (33.1)	0.63 (0.58–0.69)	0.86 (0.79–0.95)	0.95 (0.86–1.04)	0.95 (0.86–1.04)
Active (≥ 500 METs-min/wk)	4119	1340 (32.5)	0.62 (0.57–0.66)	0.80 (0.74–0.86)	0.91 (0.84–0.98)	0.90 (0.83–0.98)
**OPA**						
Inactive (0 METs-min/wk),	24,743	10,217 (41.3)	1 (reference)	1 (reference)	1 (reference)	1 (reference)
Insufficiently active (1-499 METs-min/wk)	629	241 (38.3)	0.88 (0.75–1.04)	1.07 (0.90–1.28)	1.09 (0.91–1.31)	1.10 (0.92–1.31)
Active (≥ 500 METs-min/wk)	1162	437 (37.6)	0.86 (0.76–0.97)	1.19 (1.04–1.35)	1.20 (1.05–1.37)	1.21 (1.05–1.38)
**by intensity**						
**Total PA**						
Inactive	9277	4225 (45.5)	1 (reference)	1 (reference)	1 (reference)	
Moderate to vigorous intensity	17,257	6670 (38.7)	0.75 (0.72–0.79)	0.88 (0.83–0.93)	0.95 (0.89-1.00)	
**LTPA**						
Inactive	19,795	8689 (43.9)	1 (reference)	1 (reference)	1 (reference)	1 (reference)
Moderate to vigorous intensity	6739	2206 (32.7)	0.62 (0.59–0.66)	0.82 (0.77–0.88)	0.92 (0.86–0.98)	0.92 (0.86–0.98)
**OPA**						
Inactive	24,743	10,217 (41.3)	1 (reference)	1 (reference)	1 (reference)	1 (reference)
Moderate to vigorous intensity	1791	678 (37.9)	0.87 (0.79–0.96)	1.14 (1.03–1.27)	1.16 (1.04–1.29)	1.17 (1.05–1.30)

PA, Physical activity; LTPA, Leisure time physical activity; OPA, Occupational physical activity; OR, odd ratio; CI, confidence interval. Model 1: Adjusted for age and sex. Model 2: Adjusted for model 1 plus obesity, smoking, alcohol consumption, total cholesterol, glucose, and education. Model 3: Adjusted for model 2 plus LTPA (when OPA is exposure) or OPA (when LTPA is exposure)

In the subgroup analysis by sex, active and moderate-to-vigorous intensity total PA were each associated with a lower prevalence of hypertension in women (OR 0.89, 95% CI 0.79–0.99) and (OR 0.92, 95% CI 0.85–0.99), but not in men (OR 0.93, 95% CI 0.84–1.03) and (OR 0.97, 95% CI 0.89–1.05) compared with the inactive group (*p*-value for interaction < 0.001). The ORs (95% CIs) for the prevalence of hypertension comparing active versus inactive LTPA by METs were 0.90 (0.81–0.99) in men and 0.87 (0.77–0.99) in women (*p*-value for interaction < 0.001). For OPA, the OR (95% CIs) for hypertension was not significant for the active group in men (OR 1.13, 95% CI 0.96–1.34) and women (OR 1.23, 95% CI 0.99–1.53) when compared with the inactive group (*p*-value for interaction = 0.44). Regarding intensity, moderate to vigorous intensity of LTPA was associated with a lower prevalence of hypertension in men (OR 0.89, 95% CI 0.81–0.97), but not in women (OR 0.93, 95% CI 0.84–1.03) compared with the inactive group. For OPA, the multivariable adjusted OR (95% CIs) for hypertension was significantly higher in the moderate to vigorous intensity group (OR 1.20, 95% CI 1.01–1.43) in women, but not in men (OR 1.09, 95% CI 0.95–1.26) compared with the inactive group (Table 3) (*p*-value for interaction = 0.57).

**Table 3:** Associations of total PA, LTPA and OPA with the prevalence of hypertension in men and women

Variables	n	Prevalence n (%)	Unadjusted OR (95% CI)	Model 1 OR (95% CI)	Model 2 OR (95% CI)	Model 3 OR (95% CI)	*p*-value for interaction for sex
**by METs**							
**Men**							
**Total PA**							< 0.001
Inactive (0 METs-min/wk),	3995	1897 (47.5)	1 (reference)	1 (reference)	1 (reference)		
Insufficiently active (1-499 METs-min/wk)	4212	1933 (45.9)	0.94 (0.86–1.02)	0.95 (0.87–1.04)	1.00 (0.90–1.09)		
Active (≥ 500 METs-min/wk)	3294	1314 (11.4)	0.73 (0.67–0.81)	0.87 (0.79–0.95)	0.93 (0.84–1.03)		
**LTPA**							< 0.001
Inactive (0 METs-min/wk),	7923	3764 (47.5)	1 (reference)	1 (reference)	1 (reference)	1 (reference)	
Insufficiently active (1-499 METs-min/wk)	1316	494 (37.5)	0.66 (0.59–0.75)	0.84 (0.74–0.95)	0.87 (0.76–0.99)	0.87 (0.76–0.99)	
Active (≥ 500 METs-min/wk)	2262	886 (39.2)	0.71 (0.65–0.78)	0.84 (0.76–0.93)	0.90 (0.81–0.99)	0.90 (0.81–0.99)	
**OPA**							0.44
Inactive (0 METs-min/wk),	10,550	4752 (45.0)	1 (reference)	1 (reference)	1 (reference)	1 (reference)	
Insufficiently active (1-499 METs-min/wk)	297	123 (41.4)	0.86 (0.68–1.09)	1.01 (0.79–1.29)	1.01 (0.79–1.39)	1.01 (0.79–1.29)	
Active (≥ 500 METs-min/wk)	654	269 (41.1)	0.85 (0.73–1.00)	1.10 (0.93–1.30)	1.12 (0.95–1.33)	1.13 (0.96–1.34)	
**Women**							
**Total PA**							
Inactive (0 METs-min/wk),	5282	2328 (44.1)	1 (reference)	1 (reference)	1 (reference)		
Insufficiently active (1-499 METs-min/wk)	6816	2624 (38.5)	0.79 (0.74–0.85)	0.90 (0.82–0.97)	0.94 (0.86–1.02)		
Active (≥ 500 METs-min/wk)	2935	799 (27.2)	0.48 (0.43–0.52)	0.77 (0.69–0.86)	0.88 (0.79–0.99)		
**LTPA**							
Inactive (0 METs-min/wk),	11,872	4925 (41.5)	1 (reference)	1 (reference)	1 (reference)	1 (reference)	
Insufficiently active (1-499 METs-min/wk)	1304	372 (28.5)	0.56 (0.50–0.64)	0.87 (0.76-1.00)	1.01 (0.88–1.17)	1.01 (0.88–1.17)	
Active (≥ 500 METs-min/wk)	1857	454 (24.4)	0.46 (0.41–0.51)	0.75 (0.66–0.85)	0.88 (0.77–0.99)	0.87 (0.77–0.99)	
**OPA**							
Inactive (0 METs-min/wk),	14,193	5465 (38.5)	1 (reference)	1 (reference)	1 (reference)	1 (reference)	
Insufficiently active (1-499 METs-min/wk)	332	118 (35.5)	0.88 (0.70–1.11)	1.10 (0.85–1.43)	1.16 (0.89–1.51)	1.16 (0.90–1.51)	
Active (≥ 500 METs-min/wk)	508	168 (33.1)	0.79 (0.65–0.95)	1.20 (0.97–1.48)	1.22 (0.98–1.52)	1.23 (0.99–1.53)	
**by intensity**							
**Men**							
**Total PA**							0.007
Inactive	3995	1897 (57.5)	1 (reference)	1 (reference)	1 (reference)		
Moderate to vigorous intensity	7506	3247 (43.3)	0.84 (0.78–0.91)	0.82 (0.84–0.99)	0.97 (0.89–1.05)		
**LTPA**							0.003
Inactive	7923	3764 (47.5)	1 (reference)	1 (reference)	1 (reference)	1 (reference)	
Moderate to vigorous intensity	3578	1380 (38.6)	0.69 (0.64–0.75)	0.84 (0.77–0.92)	0.89 (0.81–0.97)	0.89 (0.81–0.97)	
**OPA**							0.57
Inactive	10,550	4752 (45.0)	1 (reference)	1 (reference)	1 (reference)	1 (reference)	
Moderate to vigorous intensity	951	392 (41.2)	0.86 (0.75–0.98)	1.07 (0.93–1.23)	1.08 (0.94–1.25)	1.09 (0.95–1.26)	
**Women**							
**Total PA**							
Inactive	5282	2328 (44.1)	1 (reference)	1 (reference)	1 (reference)		
Moderate to vigorous intensity	9751	3423 (35.1)	0.69 (0.64–0.74)	0.86 (0.80–0.93)	0.92 (0.85–0.99)		
**LTPA**							
Inactive	11,872	4925 (41.5)	1 (reference)	1 (reference)	1 (reference)	1 (reference)	
Moderate to vigorous intensity	3161	826 (26.1)	0.50 (0.46–0.55)	0.80 (0.73–0.88)	0.93 (0.84–1.03)	0.93 (0.84–1.03)	
**OPA**							
Inactive	14,193	5465 (38.5)	1 (reference)	1 (reference)	1 (reference)	1 (reference)	
Moderate to vigorous intensity	840	286 (34.0)	0.82 (0.71–0.96)	1.16 (0.98–1.37)	1.20 (1.01–1.42)	1.20 (1.01–1.43)	

PA, Physical activity; LTPA, Leisure time physical activity; OPA, Occupational physical activity; OR, odd ratio; CI, confidence interval. Model 1: Adjusted for age. Model 2: Adjusted for model 1 plus obesity, smoking, alcohol consumption, total cholesterol, glucose, and education. Model 3: Adjusted for model 2 plus LTPA (when OPA is exposure) or OPA (when LTPA is exposure)

## Discussion

In this cross-sectional study based on Korean adults, our findings showed that higher levels of LTPA were associated with a lower prevalence of hypertension, whereas higher levels of OPA were associated with a higher prevalence. These associations were independent of several potential confounders as well as on mutual adjustment for each exposure independent of each other. Furthermore, our results showed that a total physical activity threshold of 145 METs-min/wk could be used to predict prevalent hypertension; however, the discriminative ability was limited; these findings are not unexpected given the cross-sectional study design which is characterised by lack of temporality. These findings contribute to our understanding of the different associations between PA domains and hypertension, supporting the PA health paradox hypothesis (Holtermann et al., 2018) and emphasizing the need to address the potential adverse health effects of occupational activities and develop interventions that promote healthier work environments.

Numerous studies have suggested that higher levels of LTPA are associated with a lower risk of hypertension [12, 13, 22], which aligns with our results We found that higher volume and moderate-to-vigorous intensity of LTPA were associated with a lower prevalence of hypertension, supporting the preventive role of LTPA in hypertension. Possible mechanisms linking LTPA and lower hypertension risk include the reduction of body weight, sympathetic activity, renin activity, insulin resistance, and improvement of vascular endothelial function and arterial stiffness.

However, the association between OPA and hypertension has produced conflicting results in previous studies. Some studies have shown an association between higher OPA and increased hypertension risk [14, 15], including a tendency for heavy occupational lifting to increase the incidence of hypertension [23]. However, these results are not universally observed [12, 16, 17]. A meta-analysis has indicated no significant association between OPA and hypertension [12]. Additionally, lower levels of OPA have been associated with a higher risk of hypertension [18, 19], while higher levels of OPA have been associated with a lower risk of hypertension [15, 17]. These discrepancies may be influenced by factors such as study populations, definitions of OPA, outcome variables, and potential confounding factors.

Sex differences may play a role in the association between OPA and cardiovascular risk [10, 11]. High OPA has been associated with an increased risk of cardiovascular disease in men [24, 25], while it has a protective effect or no association in women [25–27], although these findings are mixed. Our subgroup analyses for men and women showed that higher levels of total PA and LTPA were associated with lower prevalence of hypertension in both men and women, with slightly stronger associations for women. However, a higher OPA was associated with a higher prevalence of hypertension in women. This aligns with previous studies linking high OPA to an increased risk of heart disease in women [28]. Consistently, a prospective study indicated that heavy OPA was associated with an increased risk of new-onset hypertension specifically in women, but not in men [20]. Another study conducted among women also found a link between excessive work and the risk of hypertension [29]. These findings further support the notion that high OPA may be a risk factor for hypertension, particularly in women. The association between high OPA and a higher prevalence of hypertension in women is indeed multifactorial and may involve hormonal differences, distinct physiological response to job stressors, and additional psychosocial stressors faced by women in physically demanding occupations. Further studies are needed to clarify the possible mechanisms between higher OPA and higher risk of hypertension in women.

Several limitations should be acknowledged. Firstly, the cross-sectional design of our study limits our ability to establish causality and determine the direction of the observed associations. Future longitudinal studies would be beneficial in confirming these findings and elucidating the temporal relationship between PA domains and the development of hypertension. Additionally, as the number of participants in the insufficiently active and active OPA groups were relatively small, careful interpretation and future research with larger sample sizes are needed. Another limitation is the reliance on self-reported measures of physical activity, which are subject to recall bias and social desirability bias. Furthermore, it is hard to clarify detailed types of physical activity, such as weight-lifting and repetitive activities in static positions. Incorporating comprehensive and objective measures, such as accelerometers, in future studies would provide more accurate and reliable data on individuals’ PA levels. Lastly, our study focused on the Korean population, and caution should be exercised when generalizing the findings to other populations with different sociocultural contexts and lifestyle patterns. Further research involving diverse populations is warranted to enhance the generalizability of these findings. The strengths of this study include the use of data from the KNHANES, which is a nationally representative sample of the Korean population. Additionally, unlike previous studies that focused on either intensity or duration alone, our study considered both intensity and duration presented by METs for OPA.

## Conclusions

In conclusion, this study enhances our understanding of the association between different PA domains and hypertension risk. Higher levels of total PA and LTPA were associated with lower prevalence of hypertension in both men and women, with slightly stronger associations for women. However, higher OPA was associated with a higher prevalence of hypertension in women, highlighting the presence of sex differences in the association between OPA and the risk of hypertension. These findings underscore the importance of promoting leisure-time physical activities as a preventive measure for hypertension, while also addressing the potential negative impact of occupational physical activities on cardiovascular health. Future research should confirm these associations, explore underlying mechanisms, and develop targeted interventions that promote healthy lifestyle behaviors across different PA domains.

## Data Availability

Data sharing is not applicable to this article as no datasets were generated or analyzed during the current study.

## Abbreviations

BMI: Body mass index
CVD: Cardiovascular disease
CIs: Confidence intervals
DBP: Diastolic blood pressure
GPAQ: Global physical activity questionnaire
KNHANES: Korean National Health and Nutrition Survey
LTPA: Leisure time physical activity
MET: Metabolic equivalent of task
OPA: Occupational physical activity
OR: Odds ratio
PA: Physical activity
SBP: Systolic blood pressure
TPA: Transport physical activity
